# An overview on the treatment strategies of non-displaced femoral neck fracture in the elderly

**DOI:** 10.1186/s42836-022-00111-0

**Published:** 2022-03-01

**Authors:** Yangyang Zhou, Yuanwei Zhang, Panpan Lu, Hui Chen, Ming Ni, Yunfeng Rui

**Affiliations:** 1 Department of Orthopedics Zhongda Hospital, School of Medicine Southeast University, NO. 87 Ding Jia Qiao, Nanjing, 210003 Jiangsu China; 2 Multidisciplinary Team (MDT) for Geriatric Hip Fracture Management Zhongda Hospital, School of Medicine Southeast University, Nanjing, Jiangsu China; 3 School of Medicine, Southeast University, Nanjing, Jiangsu China; 4 Orthopaedic Trauma Institute (OTI), Southeast University, Nanjing, Jiangsu China; 5 Trauma Center Zhongda Hospital, School of MedicineSoutheast University, Nanjing, Jiangsu China; 6 Department of Orthopedics the First Medical Center, Chinese PLA General Hospital, No. 28 Fuxing Road, Beijing, 100853 China

**Keywords:** Elderly, Non-displaced, Femoral neck fracture, Conservative treatment, Internal fixation, Hemiarthroplasty

## Abstract

**Background:**

This paper aimed to review the databases on non-displaced femoral neck fractures in elderly patients. We also discussed the surgical and non-surgical treatments and selection of implants.

**Methods:**

Reviewed was the literature on non-displaced femoral neck fractures in elderly patients. Four major medical databases and a combination of the search terms of “femoral neck fractures”, “nondisplaced”, “undisplaced”, “non-displaced”, “un-displaced”, “aged”, “the elderly”, and “geriatric” were used to search the literature relevant to the topic of the review.

**Results:**

Patients who were unable to tolerate the operation and anesthesia could be treated conservatively. Otherwise, surgical treatment was a better choice. Specific surgical strategies and implant selection were important for the patient’s functional recovery.

**Conclusions:**

The non-displaced femoral neck fractures are relatively stable but carry a risk of secondary displacement. Surgical treatments may be a better option because the implants provide additional stability and allow early exercise and ambulation. Hemiarthroplasty is also an alternative for old patients with higher risks of displacement and avascular necrosis of the femoral head.

## Introduction

The femoral neck fracture is one of the most common fractures in the elderly, which seriously threatens and affects the patients’ health and quality of life [1, 2]. Currently, the optimal strategy for the treatment of non-displaced femoral neck fractures (NDFNFs) is still debated [1, 2].

The femoral neck fractures are classified into Garden type I and II (NDFNFs) and Garden type III and IV (displaced femoral neck fractures) (Table 1). The NDFNFs are prone to re-displacement, resulting in a fracture healing rate of 44.3% and a postoperative re-displacement rate of 33% to 44% [3, 4]. Therefore, surgical treatments may be a better option for the elderly [5]. Internal fixation and joint replacement are two surgical strategies, and the choice of implants and prostheses selection are at issue [6].

**Table 1:** Detailed rules of Garden classification of the femoral neck fracture

Garden type	Characteristics	Ascription
I	Incomplete fracture: The fracture line does not pass through the whole femoral neck, there is partial bone connection in the femoral neck, the fracture has no displacement, and a certain blood supply is maintained at the proximal fracture end.	Stable fracture
II	Complete fracture without displacement: Although the femoral neck was completely broken, it is well aligned. If it is a fracture under the femoral head, it may still heal, but the probability of ONFH is high. If it is a fracture of the middle or basal part of the femoral neck, the fracture is easy to heal and the blood supply of the femoral head is appreciable.	Stable fracture
III	Complete fracture with partial displacement: Mostly, the distal end of the fracture is displaced upward or the lower corner of the distal end of the fracture is inserted into the proximal section, resulting in the inward rotation and displacement of the femoral head, and the neck shaft angle becomes smaller.	Unstable fracture
IV	Complete fracture with complete displacement: The proximal end of the fracture can be rotated, and the distal end is mostly shifted back and upward. The joint capsule and synovium are severely damaged, and the blood vessels supplying the femoral head via the joint capsule and synovial membrane are also easily damaged, resulting in ONFH.	Unstable fracture

*ONFH*, osteonecrosis of femoral head

This paper aimed to review the databases on NDFNFs in elderly patients. We also discussed their surgical and non-surgical treatments and selection of implants.

## Materials and Methods

**Fig. 1:**
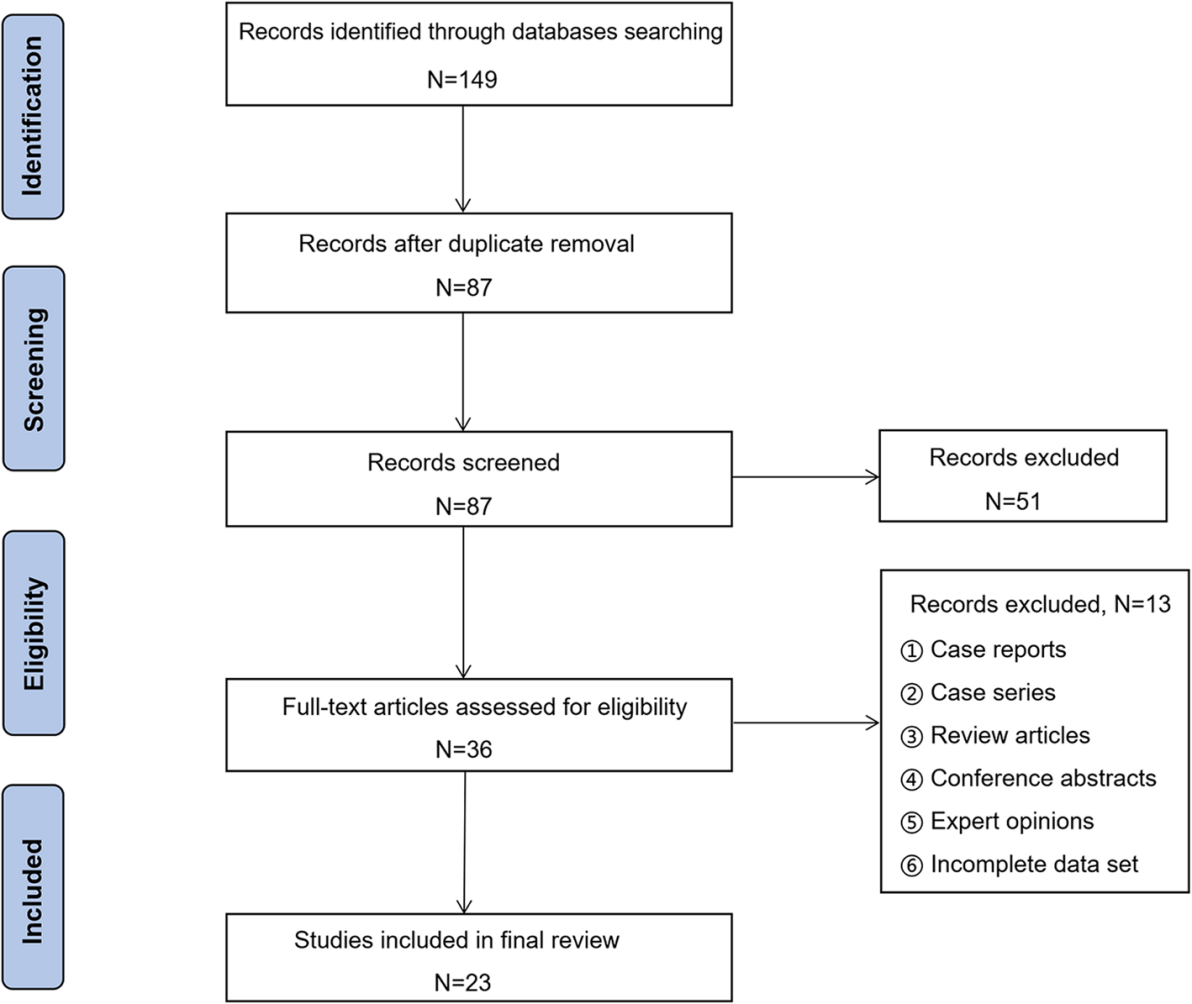
Flow chart depicting the strategy used to select the relevant research

We searched PubMed, ScienceDirect, Scopus, and Embase by using the terms “femoral neck fractures”, “nondisplaced”, “undisplaced”, “non-displaced”, “un-displaced”, “aged”, “the elderly”, and “geriatric”. All relevant titles and abstracts were reviewed. We read the full articles in the scope of the stated purposes, and the information supporting this review article was extracted. The flow chart depicting the strategy for selecting the relevant research is presented in Fig. 1.

Our inclusion criteria included: (1) clinical research; (2) patients with femoral neck fracture; (3) patients aged above 65 years old; (4) type I or II femoral neck fractures against the Garden classification; (5) clinical interventions including conservative treatments, internal fixation, and joint replacement. The exclusion criteria were: (1) duplicate publications; (2) patients with a failed internal fixation or revision operation after the initial joint replacement; (3) preoperative heart failure or mental disorder; (4) old or pathological fractures and rheumatoid arthritis; (5) the inconsistent outcome indicators.

## Results

A total of 149 articles were included in the retrieval process, and the records after duplicate removal were 87. Furthermore, after excluding the case reports, case series, review articles, conference abstracts, expert opinions and incomplete data sets, a total of 23 full-text articles were ultimately included in the final review.

### Conservative treatment

Both Garden type I and II fractures are NDFNFs. Garden type I fractures can be treated conservatively, but misdiagnosis and osteoporosis may lead to secondary displacement [7]. The complication rate of type II fractures is 1.5 times higher than that of type I fractures. In a recent retrospective study, Rzesacz *et al* [8] revealed that conservative treatments were associated with a higher complication rate as compared to surgical treatments. Therein, the incidence of complications in the conventional treatment group was as high as 30-40%. Moreover, Cserhati *et al* [9] also revealed that the complication rate, especially the osteonecrosis of femoral head (ONFH), after conservative treatment was 18.9%, which was much higher than that of surgical treatment (3.2%). Since NDFNFs also involve fracture lines, the CT scan shows the pattern of the fracture more clearly for treatment plan. In addition, compared with the use of Garden classification alone, it is more effective to combine the three-dimensional (3D) reconstruction of hip joint with Garden classification to fully evaluate the NDFNFs. With the advantages of intuitive and multiple-angle vision, 3D reconstruction can display the anatomical shape of the femoral neck and is superior to X-ray in diagnosis and displacement judgment of fracture. The 3D reconstruction of hip joint can be used to reconstruct the exact status of fracture, so as to assist the surgeons to judge the fracture type according to the Garden classification and provide scientific basis for the development of treatment plan and assessment of prognosis [10–12].

Compared to the surgical treatments, conservative treatments are associated with much more mobility restrictions, bed-related complications, family care, *etc*, which increase the total cost, morbidity, and mortality [8, 9, 13].

### Surgical treatment

Surgical treatments mainly include internal fixation and joint replacement (especially hemiarthroplasty or total arthroplasty), which are systematically reviewed and summarized in the following sections.

### Internal fixation

#### Timing of internal fixation

Kim *et al* [14] studied the timing at which patients underwent the internal fixation. Among 58 patients with a mean age of 74 years, the 1-year mortality rate in the patients who had undergone the internal fixation within 24 h after admission was only 4.7%, which was lower than in patients undergoing internal fixation 24 h after admission (13.3%). The 1-year mortality rate was 7% in patients who underwent the internal fixation within 48 h after the admission, and 72% of the patients recovered their walking ability to their pre-fracture level. Thus, an internal fixation performed shortly after admission (within 48 h after admission) may achieve better clinical outcomes and have fewer complications, such as nonunion and late segmental collapse of the femur.

#### Implant Selection

Many implants are used for treating NDFNFs, including cannulated screws, cancellous bone screws, dynamic hip screws, targon system, emerging dynamic locking plates, and full-thread headless compression screws. The posterior retroversion angle is closely related to the prognosis of the patients with NDFNFs. The retroversion of femoral head and frequently associated comminution of the posterolateral wall compromise blood supply of the femoral head [15, 16]. Palm *et al* [17] reviewed 113 patients with NDFNFs. One year after surgery, 25 patients (22%) had a posterior retroversion angle of femoral head more than 20°, and the failure rate of internal fixation was 56%; 78% of patients had a posterior retroversion angle less than 20°, and the failure rate of internal fixation was 14%. The increased posterior retroversion angle is a risk factor for the re-operation of femoral neck fractures. Besides, Yamamoto *et al* [18] also suggested that some NDFNFs on X-ray may combine with the posterior retroversion angle of more than 20° on CT images. The posterior retroversion angle of more than 20° is intimately related to the poor prognosis of femoral neck fractures.

Cannulated screw is one of the ideal choices for the fixation of NDFNFs in elderly. Chen *et al* [19] treated 37 patients (over 80 years old) with NDFNFs using cannulated screws. Two years after surgery, the total healing rate was 95%, and the total success rate was 84%. The main reasons for revision were the failure of internal fixation, nonunion of fracture, and ONFH [20, 21]. Moreover, the high frequency of revision was also related to the factors such as poor bone quality in the elderly, severe osteoporosis, and surgical techniques [22].

Fixation with the cancellous bone screw is also one of safe and effective surgical approaches. Lee *et al* [23] conducted a retrospective study on 116 elderly patients who were treated with cancellous bone screws. Two years after surgery, 85% of the patients recovered to pre-injury activity level, and 90% of patients had no pain. In a retrospective study, Manohara *et al* [24] found that 90% of patients could independently walk postoperatively, 20% of patients felt pain during exercise, and the nonunion rate was 3%. Chiu *et al* [25] also showed that the non union rate of NDFNFs in patients treated with cancellous bone screws was only 2%.

Dynamic hip screws effectively treat NDFNFs with few complications and a low re-operation rate. Compared to the cannulated screws, patients treated with dynamic hip screws had a higher Harris score one year after surgery and a lower re-operation rate [25, 26]. Watson *et al* [27] conducted a comparative study on 62 patients treated with dynamic hip screws and cancellous bone screws, and there were six deaths (19.3%) in both groups. The re-operation rate (3.2%) was lower in the dynamic hip screw group than in the cancellous bone screw group (10.3%). In addition, Makki *et al* [28] studied elderly patients aged 70 on average and found that compared to the dynamic hip screws alone, dynamic hip screws combined with anti-rotation screws may not reduce the incidence of ONFH and revision rate.

Recent studies have revealed that those systems were associated with a low incidence of postoperative complications in NDFNFs [29]. Compared to traditional screws, the targon system is associated with a lower revision rate and prevents femoral neck-head fragment settlement. Moreover, compared to the cannulated screws (48.8%), the revision rate of targon system within the first postoperative year is 4.7% [30]. Compared to the dynamic hip screws, the targon system is associated with low rates of femoral neck-head fragment settlement and fewer hemiarthroplasty due to fixation failure [31]. In addition to the targon system, the emerging dynamic locking plate is also widely used. Van Walsum *et al* [32] used emerging plates for NDFNFs and found that only 6 cases failed among 149 cases.

Most patients experienced the postoperative femoral neck shortening, resulting in the varus hip and poor gait function. Chiang *et al* [33] treated 50 NDFNFs using partially-threaded cannulated screws and full-threaded headless compression screws. The two implants provide stable fixation but cannot prevent femoral neck shortening and varus displacement.

#### Dynamic magnetic resonance imaging (MRI) and surgical selection

In NDFNFs, nonunion and late collapse of the femoral head are the two major complications. Patients aged 69 or younger have a high risk of ONFH after percutaneous screw fixation [34]. Moreover, preoperative traction also increases the risk of postoperative ONFH [35]. The blood supply to the femoral head is a significant factor affecting fracture healing. Morimoto *et al* [36] examined blood perfusion of the femoral head using dynamic MRI enhanced integral color imaging and classified the perfusion states into four types. With type A, the color of fracture area was consistent with that of the healthy side, indicating that its blood perfusion was the same; with type B, the color of the fracture area was darker than the femoral head area of the healthy side, indicating that the blood perfusion was reduced; with type C, the fracture area was black, indicating that there was no perfusion in this area. When they treated their patients with three cannulated screws, the nonunion rates of type A, B, and C were 0, 6.7%, and 50.0%, respectively, and the collapse rates of the femoral head were 0, 4.4%, and 0, respectively. For type C blood perfusion, joint replacement should be the first option, and a revision operation should be avoided if all possible.

### Hemiarthroplasty

In recent years, hemiarthroplasty is increasingly performed in elderly NDFNFs. According to the statistics of the Norwegian fractures data center, NDFNF patients treated with hemiarthroplasty rose from 2.1% in 2005 to 9.7% in 2014 [37].

Although different types of internal fixation have been widely acknowledged in the treatment of NDFNFs, Lin *et al* [38] found that the revision, nonunion and delayed union rates were higher in patients over 75 years old with femoral neck fracture receiving internal fixation than in those with hemiarthroplasty, and the prognosis was also worse. Lu *et al* [39] conducted a randomized controlled trial comparing hemiarthroplasty and internal fixation for NDFNFs in 78 patients (aged 85 to 100 years). The re-operation rate of the hemiarthroplasty was significantly lower than that of internal fixation (5.41% *vs.* 21.4%). Only the surgical methods had a significant impact on the occurrence of re-operation as shown by a Cox proportional hazard regression model. There were no significant differences between the two treatments in survival time and mean Harris score 5 years after surgery. However, hemiarthroplasty resulted in a significantly higher excellent-to-good rate. In addition, given nearly one-third of the elderly patients have combined dementia, following the postoperative rehabilitation instructions is difficult. Therefore, internal fixation may be a better choice in these patients [40].

Regarding this, Olofsson *et al* [41] conducted a retrospective study including 180 elderly patients (all aged above 70 years) with NDFNFs and dementia. They found no difference in the incidences of complications and mortality at 4 months and 1 year of follow-ups after the internal fixation and hemiarthroplasty. Dementia may not be a contraindication for hemiarthroplasty. Moreover, because the artificial femoral heads gradually degenerate, the postoperative survival of the autologous femoral head may be longer [42–44]. Currently, however, no study has compared the long-term outcomes between artificial and autologous femoral heads.

### Controversies on the choice of surgical methods

Currently, the optimal treatment for NDFNFs is still controversial because few RCTs with a high level of evidence were conducted. The major considerations include postoperative function, complications, reoperation rate, and total cost [26, 34, 45–47]. You* et al* [48] indicated that internal fixation was associated with mild pain and better patient satisfaction but was associated with a high re-operation rate (20%). Moreover, hemiarthroplasty was a cost-effective option compared to internal fixation ($23,467 *vs.* $25,356) [47]. Hemiarthroplasty was also associated with less complications, lower re-operation rate, and better early functional recovery [46]. However, the downsides include longer operative time, more intraoperative blood loss, and higher intraoperative risk [48–50]. Dolatowski* et al* [26] conducted an RCT and revealed that hemiarthroplasty didn’t outperform internal fixation in the re-establishment of the postoperative hip function. However, we believe that, with rapid development of IT technologies, 3D printing, computer-assisted navigation and other new technologies will find their application in orthopedic practice [51–56]. 3D printing-related personalized technology and the computerized navigation might offer effective solutions to the selection of surgical methods for the NDFNFs in the elderly.

## Conclusions and perspectives

The NDFNFs are relatively stable but carry a risk of secondary displacement. Surgical treatments may be a better option because the implants provide additional stability and allow early exercise and ambulation. Hemiarthroplasty is an alternative treatment for elderly patients with higher risks of displacement and avascular necrosis of femoral head.

## Data Availability

The datasets used and/or analyzed during the current study are available from the corresponding author on reasonable request.

## Abbreviations

NDFNFs: Non-displaced femoral neck fractures
ONFH: Osteonecrosis of femoral head
MRI: Magnetic resonance imaging
3D: Three-dimensional
RCTs: Randomized controlled trials
IT: Information technology
